# Evaluating Crop Area Mapping from MODIS Time-Series as an Assessment Tool for Zimbabwe’s “Fast Track Land Reform Programme”

**DOI:** 10.1371/journal.pone.0156630

**Published:** 2016-06-02

**Authors:** Konrad Hentze, Frank Thonfeld, Gunter Menz

**Affiliations:** 1 Remote Sensing Research Group, Department of Geography, University of Bonn, Bonn, Germany; 2 Center for Remote Sensing of Land Surfaces, University of Bonn, Bonn, Germany; University of Maryland at College Park, UNITED STATES

## Abstract

Moderate Resolution Imaging Spectroradiometer (MODIS) data forms the basis for numerous land use and land cover (LULC) mapping and analysis frameworks at regional scale. Compared to other satellite sensors, the spatial, temporal and spectral specifications of MODIS are considered as highly suitable for LULC classifications which support many different aspects of social, environmental and developmental research. The LULC mapping of this study was carried out in the context of the development of an evaluation approach for Zimbabwe’s land reform program. Within the discourse about the success of this program, a lack of spatially explicit methods to produce objective data, such as on the extent of agricultural area, is apparent. We therefore assessed the suitability of moderate spatial and high temporal resolution imagery and phenological parameters to retrieve regional figures about the extent of cropland area in former freehold tenure in a series of 13 years from 2001–2013. Time-series data was processed with TIMESAT and was stratified according to agro-ecological potential zoning of Zimbabwe. Random Forest (RF) classifications were used to produce annual binary crop/non crop maps which were evaluated with high spatial resolution data from other satellite sensors. We assessed the cropland products in former freehold tenure in terms of classification accuracy, inter-annual comparability and heterogeneity. Although general LULC patterns were depicted in classification results and an overall accuracy of over 80% was achieved, user accuracies for rainfed agriculture were limited to below 65%. We conclude that phenological analysis has to be treated with caution when rainfed agriculture and grassland in semi-humid tropical regions have to be separated based on MODIS spectral data and phenological parameters. Because classification results significantly underestimate redistributed commercial farmland in Zimbabwe, we argue that the method cannot be used to produce spatial information on land-use which could be linked to tenure change. Hence capabilities of moderate resolution data are limited to assess Zimbabwe’s land reform. To make use of the unquestionable potential of MODIS time-series analysis, we propose an analysis of plant productivity which allows to link annual growth and production of vegetation to ownership after Zimbabwe’s land reform.

## Introduction

After Independence in 1980, Zimbabwe had to redress its screaming injustice in wealth and land ownership. More than half of the country's arable land was held by less than 7,000 commercial—often white—farmers, while the majority of the population was concentrated on the other, overall less productive half [1]. The newly elected democratic government started a carefully arranged land reform program which aimed to redistribute commercial farmland, but it did not meet its self-set ambitious goals [2]. As one consequence of the perception of a failed land reform program, numerous heterogeneous groups of people started to invade commercial farms and to disseize their owners in the year 2000 [3]. In the following period, the agricultural sector of Zimbabwe experienced drastic changes. The consequent vivid debate about whether or not this almost fifteen year-old Zimbabwean “fast track land reform programme” (FTLRP) was successful, has still not come to an end [4,5]. Controversy is ongoing whether overall goals of the program have been met or not and which goals outweigh others. A general challenge in land reform assessments lies within the fact that redistribution processes can be evaluated with different criteria [6,7]. Among the discussed measures, the state of national agricultural production patterns recognizes significant attention. But up to date, spatial explicit, objective, and country-wide datasets on the spatio-temporal development of agricultural area in Zimbabwe are rare [8]. Throughout the discourse on the success of Zimbabwe’s land reform, a general need of reliable, spatial data becomes apparent. A systematic literature review carried out in a previous study reveals a significant lack of recent representative statistical and quantitative spatial data in scientific publications from all research perspectives [6]. Authors themselves highlight the absence of spatial data on relevant issues connected to the FTLRP [9] which can be linked to untapped potential of innovative geospatial methods [6].

Remote sensing analysis offers a number of different approaches to deliver spatial, reproducible data on land use and land cover (LULC) and its change. For more than three decades, various methods of remote sensing have been developed to determine plant conditions and vegetation characteristics. Indices such as the Normalized Difference Vegetation Index (NDVI) relate reflectance values to vegetation cover or above ground biomass by making use of the specific relationship between the ratio of red and near-infrared reflectance and plant status [10]. It allows determination of the fraction of photosynthetic active radiation (FPAR) from remote sensing data and hence to calculate information on plant cover such as the Leaf Area Index (LAI) while taking background, atmospheric, and bidirectional effects into account [11]. NDVI and other indices have successfully been applied to gather agricultural information at different scales [12,13]. Among these methods, time-series analysis has been used extensively to map plant production and LULC information. Applied in different regional contexts, time-series analysis has shown promising results in differentiation of land cover types such as cropland and, under specific conditions, also different crop types using measures of phenology [14,15]. This has been successfully proven in Southern Africa and also in Zimbabwe [16]. Phenology describes the seasonal plant cycle and its characteristic stages such as green-up onset, peak of greenness, start of senescence, or length of vegetative season [17]. As different plants have distinct phenological characteristics, they can be identified and classified according to these temporal measures which have to be acquired throughout a phenological cycle.

We argue that this potential of remote sensing has not been tapped sufficiently to deliver input into the assessment of Zimbabwe's land reform. The goal of this study was to evaluate whether time-series analysis as a method of spatio-temporal mapping is capable to determine changes in Zimbabwe’s agricultural area which took place after the FTLRP between 2001 and 2013. It therefore aimed to fill the previously identified gap of spatial objective data within the discourse on the success of Zimbabwe's land reform.

Recent work on time-series and FTLRP has assessed productivity within an agricultural area extracted from external land use datasets [13,18,19]. We tested whether coarse-scale, multi-temporal data can be used to classify LULC and its changes occurring as a consequence of the FTLRP in Zimbabwe. If crop area could be mapped on a yearly basis with a reproducible method, an important aspect of land reform assessment could provide meaningful objective input to a highly politicized discourse [6]. Changes and volatility of land use in former commercial farmland were to be mapped because titles and mode of agricultural production shifted drastically in this region.

For Zimbabwe, with malnourishment being prevalent in many parts of the country [20], spatial accurate information on agricultural production and fallow land is key. More accurate information would not only lead to better assessments of the FTLRP, but could also indicate areas where farmers are in need of extension services, training and financial support. This has been formulated as central to improve nationwide productivity of redistributed farm areas [21]. LULC information has been identified as one of the most important variables for a variety of societal aspects in a recent Global Earth Observation System report [22].

Within the current study, emphasis was put on the ability of Moderate-Resolution Imaging Spectroradiometer (MODIS) NDVI imagery and derived phenological parameters to map cropland area over twelve agricultural seasons in thirteen years (2001–2013) with explicit focus on redistributed farmland. MODIS offers several advantages for these efforts: In addition to the robustness to cloudiness, an analysis using one methodology applied to one sensor at regular acquisition dates avoids incomparable results due to different acquisition dates or small study areas. Existing LULC datasets for the region have been based on labor intensive Landsat classifications; updates of cropland distribution would require the manual processing of several cloud free scenes per year.

Our specific research questions were:

- How accurate can time-series analysis of MODIS data map Zimbabwe's redistributed agricultural area?
- Can MODIS data be used to map changes and volatility of agricultural area in the time frame 2001–2013?
- Can the generated spatial products be linked to information on land tenure to correlate information on LULC change with information on tenure change?

## Study Area

Zimbabwe, bordered by the rivers Limpopo (South) and Zambezi (North) and a mountain range in the east, is a landlocked country in the torrid zone with a size of 390,757 km^2^ and about 13 million inhabitants [23]. The country's agro-ecological conditions are characterized by contrasting climatic and soil conditions. The average rainy season lasts for about 4 months (November—March), limiting the access to qualitative satisfactory optical remote sensing data of high resolution. During the austral winter, stable air layers are caused by sinking masses of stronger trade winds and hinder convection. During the rainy season, trade winds become weaker due to low pressure areas over the land surface and lead to unstable conditions favoring convective currents [24]. However, droughts are regular experiences and form one of the many obstacles of newly resettled farmers in addition to the instability of markets as a consequence of economic recession. Generally, the fertile and climatic favorable tsetse-free highland with highly suitable areas for crop production can be distinguished from the less productive lowland [25]. Freehold commercial farmland was situated on these fertile areas, whereas communal areas for indigenous people were found on less fertile land. Precipitation ranges from 400 mm to 2,500 mm with a south-west to east gradient [26]. Elevation in the eastern mountains ranges up to 2,500 m above sea level, while the central plateau, known as 'highveld' has an approximate height of 1,200 m above sea level. With parent material being the most important soil building factor, soils are characterized by low nutrition values and rapid degradation [27]. They are a major limiting factor for agricultural production and natural plant growth, main soils groups include *Arenosols*, *Cambisols*, *Leptosol*, *Lixisols* and *Luvisols [28]*. The vegetation of Zimbabwe is partially comprised of grassland (*Themeda*, *Hyperrhenia*, *Loudetia*), thicket (*Combretum*) shrub-savanna (*Colophospermum*), and afromontane forests (*Pittosporum*, *Maranthes*). Much of the country is covered by tree-savanna (*Terminalia*, *Burkea*, *Baikiaea*) and miombo woodland (*Brachystegia*, *Julbernardia*) [29,30].

**Fig 1:**
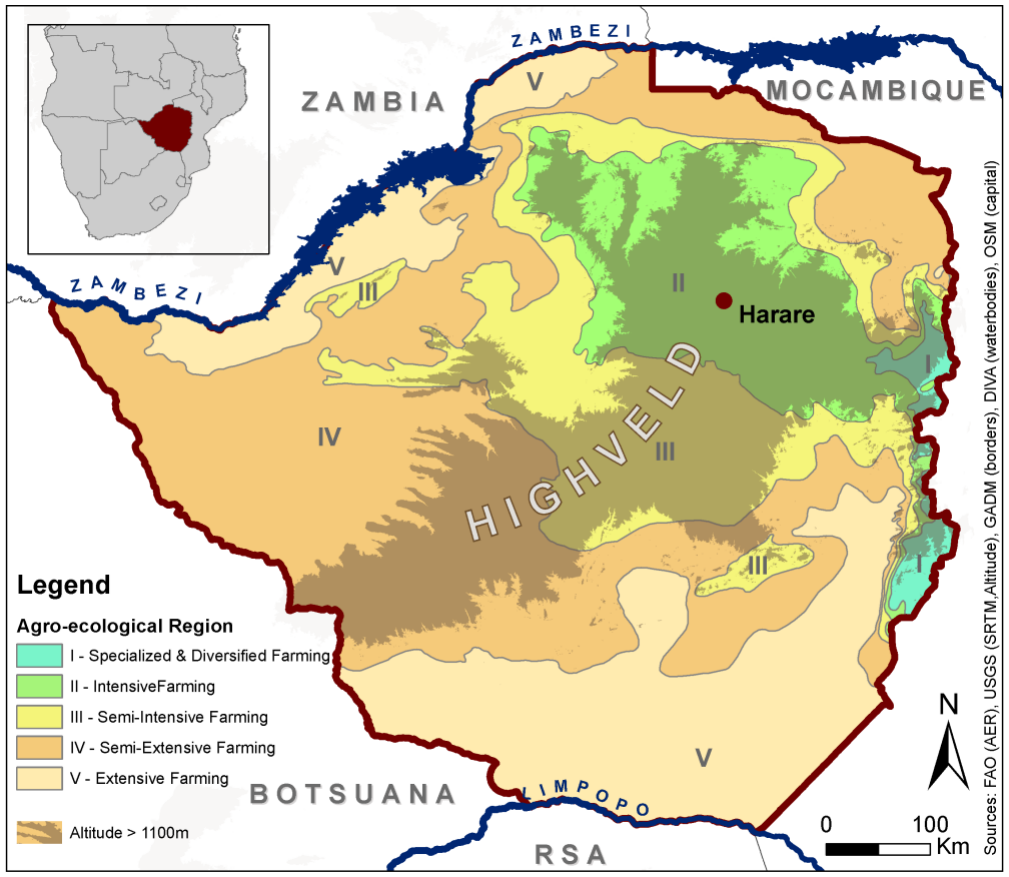
Agro-ecological regions of Zimbabwe.

Zimbabwe can be stratified into six different agro-ecological regions. They are related to climatic and soil conditions and therefore indicate the suitability for rainfed agriculture [31]. To avoid negative impact of different climatic conditions, we restricted our analysis to agro-ecological region II (AER II, see Fig 1) which is situated at the northern part of the 'highveld' and highly suitable for crop production. This zone is also characterized by a heterogeneous land tenure pattern including different types of resettlement schemes, communal areas and large scale commercial farming, allowing a spatial correlation of land tenure and classification results. Our study was restricted to redistributed former freehold land within AER II.

With more than 20% share, agriculture forms a major contributor to the national GDP [32]. In the past, Zimbabwe underwent a number of political and socio-economic changes in addition to the thorough land reform program: As Rhodesia from colonial style-rule by British South Africa Company (BSAC) to self-declared independence, later from guerrilla war to official independence and majority rule as Zimbabwe [33]. Twenty years after independence, the country was hit by an economic recession and hyperinflation. Currently, in 2016, the economy has partly recovered under 'the government of national unity' which followed the 'Global Political Agreement' and was formed in 2009 by the three major parties ZANU-PF, MDC-T and MDC-M. The dollarization and abolishment of the Zimbabwean Dollar in 2009 lead to further stability [34].

## Low Resolution Multi-Temporal Data as a Basis for LULC Classification

The increasing demand for LULC information on regional scales is one driver of the growing number of coarse-scale remote sensing-based classifications [35]. These datasets are often based on multi-temporal imagery which are acquired with high repetition rates. Products of multi-temporal imagery such as NDVI are available in continuous intervals which allow the analysis of phenological information, since regular NDVI and reflectance values can be used to construct curves which represent the phenological activity of the land surface. Well-established methods to interpolate the data include the Harmonic Analysis of time-series (HANTS), Best Index Slope Extraction (BISE), function-based curve fitting approaches or smoothing of temporal signal such as the Savitzky-Golay filtering [36]. These methods generate continuous, temporal time-series profiles on a per pixel basis which can be analyzed and described through a set of specific parameters.

Classifications of multi-temporal satellite based information form the basis for a majority of global LULC datasets. Hence, time-series can be considered as an extensively explored, applied, validated, and improved method of remote sensing analysis. Global LULC datasets, such as IGPB-landcover [37], MODIS [38], or GlobCov [39] are widely used in studies of different fields, often with the assumption of accuracy and applicability for the specific research question. However, critical reviews and efforts to assess comparability of results have shown that global LULC datasets have to be treated with great caution, and that methods of specific datasets incompletely match research designs and questions [40,41]. Fritz et al. provide a detailed comparison of global and regional land cover maps with an explicit focus on the agricultural domain in Africa [42]. They have compared 16 national figures (including Zimbabwe) for agricultural area from different datasets with FAO statistical data. Table 1 shows their results with figures of deviation which exemplify that also MODIS, with its relatively high spatial resolution, has strong limitations for global applications.

**Table 1:** RMSE of cropland area comparing different land cover products to national^1^ FAO statistics [42].

Land cover type	RMSE(km²)
GLC-2000 minimum^2^[43]	21,064
GLC-2000 maximum	76,802
SAGE[44]	25,109
MODIS minimum[45]	27,787
MODIS maximum	36,504

^1^ For Botswana, Burkina Faso, Central African Rep., Chad, Eritrea, Gambia, Lesotho, Mali, Mauritania, Morocco, Namibia, Rwanda, Senegal, Somalia, Togo, Zimbabwe

^2^“Maximum” and “minimum” refer to in- or exclusion of mixed classes

**Fig 2:**
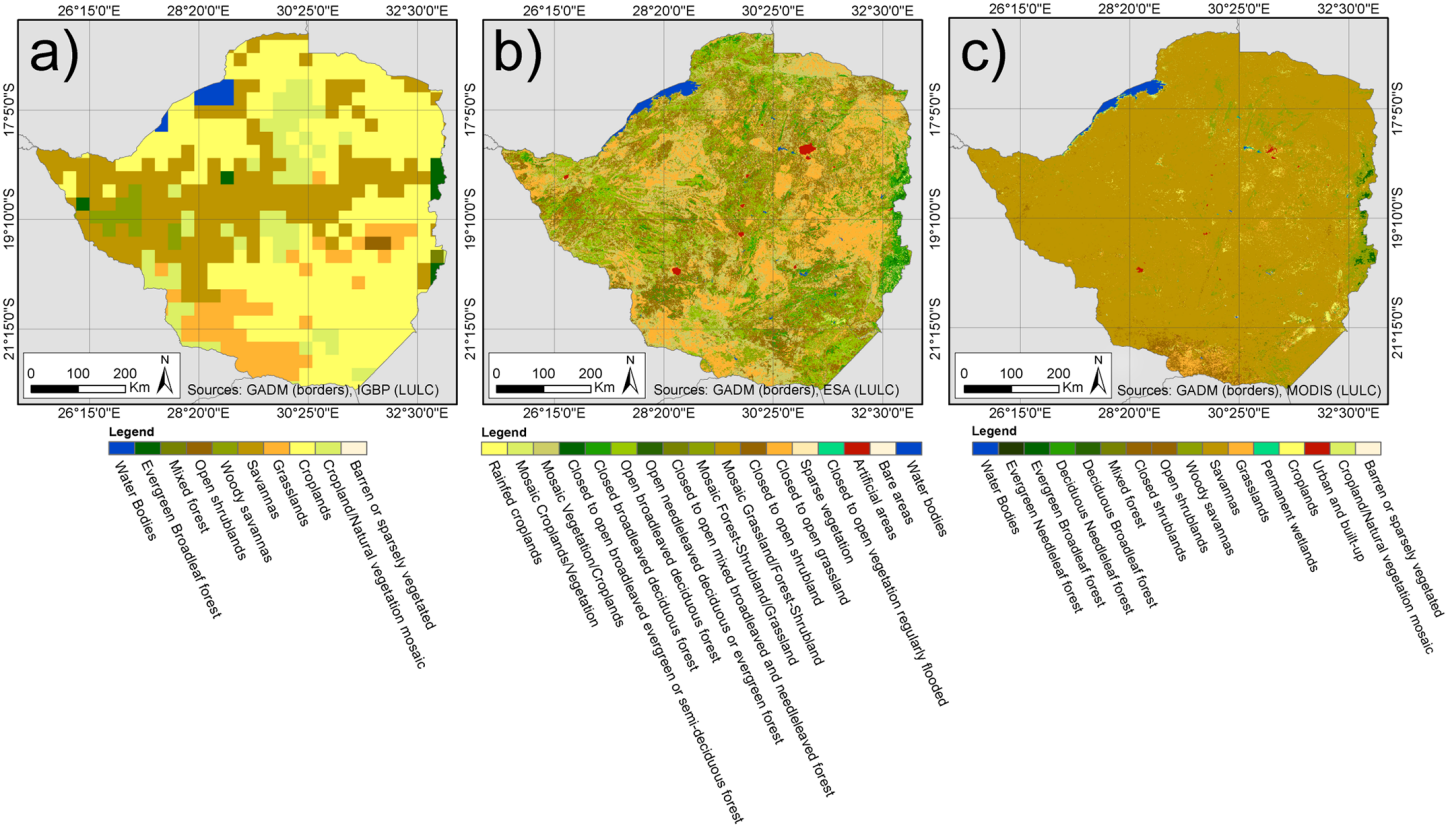
Comparison of different LULC classifications and class definitions: (a) IGBP, 1993, (b) GlobCover, 2009(c) MODIS13Q1, 2014 (Band1).

As a consequence, datasets have to be chosen with care and criteria for selection have to be made explicit. Also, caution has to be applied to the interpretation of LULC classes of global datasets, because they often follow different classification schemes (Fig 2). Especially, classes with mixed ground cover, for example Savannahs, are extremely inconsistent in terms of variables such as ground cover threshold. Low producer and user accuracies for these LULC types are reported for different classification schemes [35]. A major source of inaccuracies is the fact that global products have to stratify land surface characteristics over large areas of different latitude, altitude, and climatic regimes which leads to heterogeneity and low significance of classes.

Other limitations of global products arise from sensor specifications. The Advanced Very High Resolution Radiometer (AVHRR) data is popular for its long availability time frame and its consistent quality. However, its spatial and spectral resolutions lead to difficulties in classification accuracies (Fig 2a). With a ground resolution of 1x1 square kilometers, AVHRR datasets face problems of mixed pixels. More recently, especially for smaller scale regional analysis, imagery from MODIS is used, because it combines suitable temporal, spectral and spatial resolution [11]. The sensors of Terra and Aqua have been operating since 1999 and 2002, respectively [46] and show major improvements compared to previously-used systems, such as sensitivity to crop area. Pittman et al. conclude from their global cropland mapping based on MODIS, that crop NDVI phenology varies greatly on global scale and that regional studies based on this sensor will lead to improved results [47].

For the discrimination of agricultural land, several studies using MODIS show promising results on a global and regional level [38]. In Zimbabwe, nationwide production anomalies [13], yield predictions [48] and also hard classifications of crop type were carried out successfully using time-series analysis [16,49]. Acknowledging this potential of multi-temporal data and analysis, but also the limitations in contexts of specific research questions, we tested the suitability of MODIS data for crop classifications as an FTLRP assessment tool in a clearly defined study area with rather homogeneous climatic conditions and land tenure.

## Data and Preprocessing

Five type of spatial datasets were used for this regional cropland mapping: MODIS NDVI (1) and RED/NIR (2) composites for a time-series analysis for the whole study period of 12 seasons (2001–2013); Landsat scenes (3) for construction of endmembers based on supervised classification; as well as an external LULC data set (4) providing further information as input and validation data. Additionally, we made use of Google Earth imagery (5) to verify different calculation results.

### MODIS data

**Fig 3:**
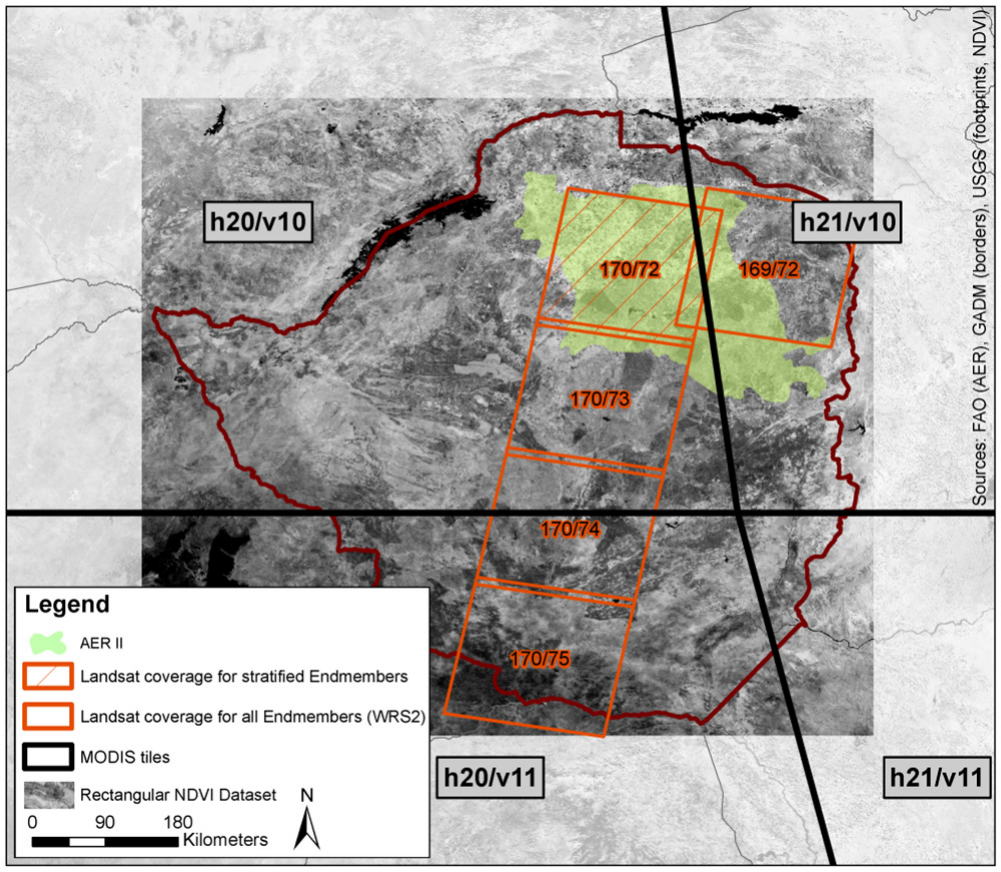
MODIS and Landsat tiles used in this study. Landsat tile used for the creation of agro-ecological stratified endmembers is highlighted. The rectangular NDVI dataset represents the extent of the NDVI dataset.

To cover the entire area of Zimbabwe, we mosaiced four tiles of the MODIS product MOD13Q1 (h20v10, h21v10, h20v11, h21v11) which we acquired through the United States Geological Survey (USGS) reverb tool [50]. MOD13Q1 is a 16-day maximum value composite (MVC) product of different vegetation and quality indices, and spectral bands, with an annual sequence of 23 scenes per calendar year [51]. The NDVI layer (1), as well as RED (4) and NIR (5) were extracted and reprojected from Sinusoidal (SR-ORG:6965) to WGS84 (EPSG:4326) and clipped to a rectangular extent on a pixel based method to avoid spatial shifts (Fig 3). We did not extract the quality layer (3) since we applied filtering at a later stage, a method of removing noise in time-series [52]. For a better overview of the interannual spatio-temporal conditions within the study area and period, and also to adjust TIMESAT calculations, an average NDVI year was produced. Therefore, we calculated the mean for each of the 23 time steps based on the information from thirteen years. To avoid generalization, the data was not used for data gap filling. Missing or faulty scenes were replaced with mean values from preceding and subsequent scenes.

### Landsat data

Landsat imagery was accessed through EarthExplorer and GLOVIS from USGS [53,54]. To retrieve the reference data for MODIS LULC classifications, we chose a year with high coverage of Landsat scenes (Level1) for two day-of-year (DOY) time steps, representing the rainy season in February and the dry season in September. We downloaded all scenes regardless of cloud cover and set a maximum deviation of 8 days from the mean DOY acquisition date of the acquisition clusters to consider the scenes as one time step. For this year, 2005, we mosaiced all scenes with all bands for both time steps, resulting in two almost country-wide datasets. We used these mosaics for general orientation and extracted 5 cloud-free scenes (Fig 3) for the creation of training and validation datasets (5.1).

### Auxiliary spatial data

As an additional source of information for training and validation of classifications, the regional SADC (Southern African Development Community) LULC data set was acquired and clipped to the national boundary from the GADM database of global administrative areas [55]. This data set was produced by different institutions and based on different data sources. For Zimbabwe, the data was published by the local Forestry Commission, together with the German Technical Cooperation (GTZ). Landsat Scenes were interpreted manually and resampled to coarser scale, the production date was specified as 1997 [56]. To account for the large time lag, the SADC LULC data set was only used for contextualizing and cross-checking of classification results and input parameters.

Land tenure data for the region was acquired from the Foreign Agricultural Service of the United States of America (FAS). This data classifies farm structures in communal, freehold and state land and provides additional information on land holdings after the land reform program. It was considered as accurate in other studies [18].

## Methods

In order to derive precise information about LULC and its change in the years 2001–2013, three consecutive methods were applied in this study. *First*, pre-processed MODIS imagery was enhanced and a time-series as well as seasonal parameters (SP) were calculated and stacked (5.1). *Secondly*, we constructed endmembers of LULC classes based on Landsat data which we classified with support vector machines (SVM) and upscaled to MODIS resolution (5.3). Finally, the endmember dataset was used to run a random forest classification (RF) on the MODIS data stacks (5.4), the yearly results were reclassified to binary yearly datasets (rainfed/non-rainfed).

**Fig 4:**
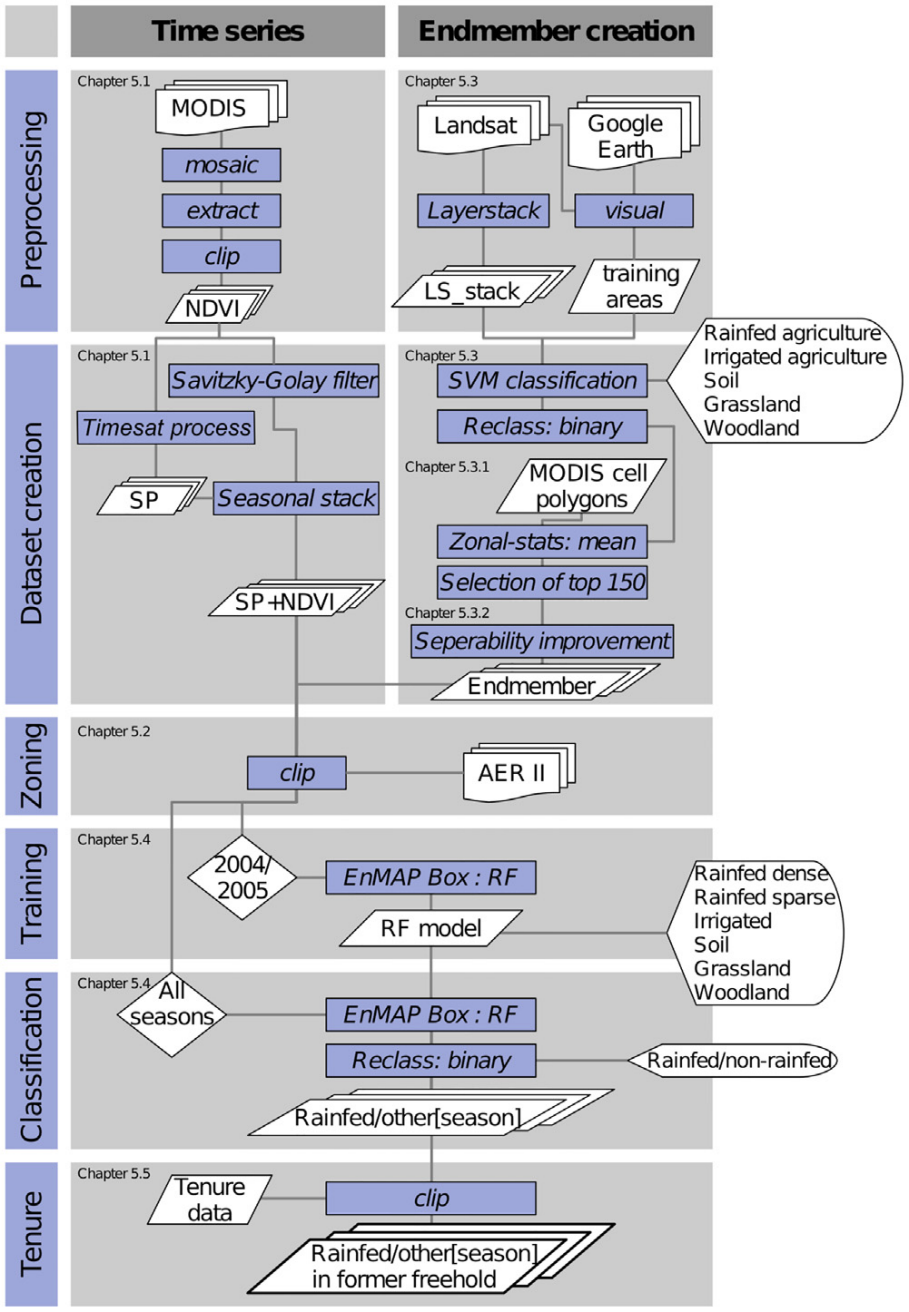
Flowchart of the processing chain for LULC classification.

To assess the regional applicability of these methods, we required the need of an agro-ecological stratification (5.2), as well as an improvement of class separability (5.3.2). Furthermore, the final binary results were extracted to former freehold tenure as the region of interest (5.5). This workflow is visualized in Fig 4.

### Time-series analysis

Applying the software TIMESAT on the average phenological year, parameters for the smoothing algorithm were tested and used for further analysis. A phenological year is characterized by seasonal plant activities and does not have to relate to the calendar year. TIMESAT provides different methods to reduce noise and to smooth the temporal curve as well as the option to derive SP [57].

**Fig 5:**
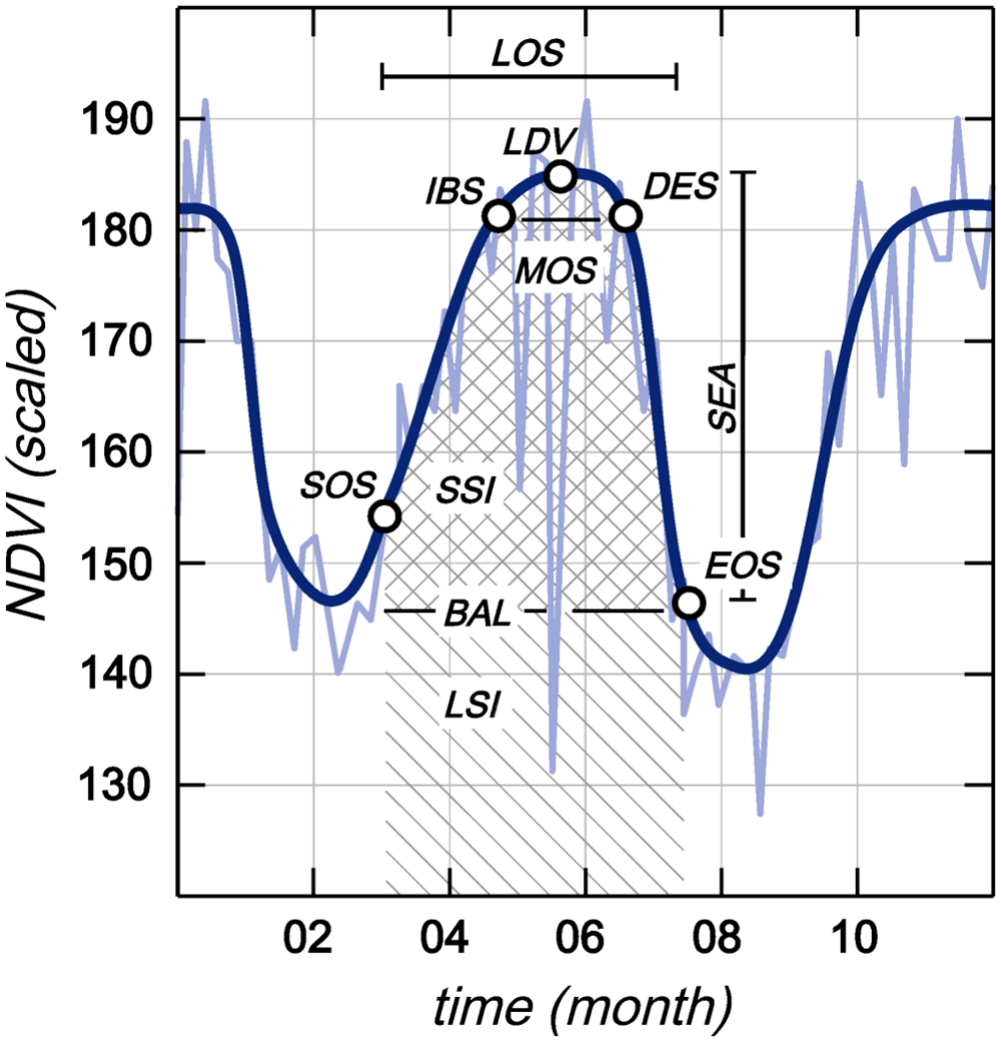
Exemplary visualization of an original and a filtered NDVI-curve, as well as 11 Seasonal Parameters. Adapted from [59].

A Savitzky-Golay filtering was chosen to smooth the NDVI curve of the time-series. This method ensures a high locality by using a moving window which replaces values by new smoothed values, derived from neighboring values [58]. Testing showed best results for a spike-method with value 2 to calculate 11 seasonal parameters:

1. *Start of Season* (**SOS**), which we defined as the increase of NDVI of the fitted function to 0.2 above the minimum. It is located on the left side of the yearly maximum.
2. *End of Season* (**EOS**), which we defined as the decrease to 0.2 above the minimum following the peak of the function.
3. *Length of Season* (**LOS**), the time between the SOS and EOS.
4. *Base Level* (**BAL**) is the average of the minimum values left and right of the season.
5. *Mid of Season* (**MOS**) is computed as the mean of times where 80% values occur left and right of the absolute maximum of the NDVI function.
6. *Largest Data Value* (**LDV**), the absolute highest single value in the whole season.
7. *Seasonal Amplitude* (**SEA**), which is the difference between the LDV and the BAL.
8. *Increase at Beginning of Season* (**IBS**), the calculated ratio between the first 20% and 80% value (left) of the fitted function (in Fig 5 between SOS and IBS).
9. *Decrease at End of Season* (**DES**), the absolute between the second 80% and 20% value (right) of the fitted function (in Fig 5 between EOS and DES).
10. *Large Seasonal Integral* (**LSI**), the area under the NDVI curve between SOS and EOS.
11. *Small Seasonal Integral* (**SSI**), the area of the difference between LSI and BAL between SOS and EOS [59].

We then created two types of composites (C_1_ and C_2_) for each phenological year (PY_i_). C_1_ with a specific amount of layers consisting of NDVI composites (MVC) and seasonal Parameters (SP), C_2_ with (RED) and (NIR) added. Classifications were run on both composite types, and accuracies were compared to evaluate whether the addition of reflectances improves classification results. Because the phenological year does not match the calendar year on the southern hemisphere, we created composites according to SOS and EOS of the calculated mean year.

$$C_{1}{PY}_{i}\;=\;({MVC}_{\left(i-1\right)}13-23\sim {MVC}_{\left(i\right)}1-12)+{SP}_{i}\left[{SOS}_{i},{EOS}_{i},\cdots ,{LSI}_{i},{SSI}_{i}\right]$$

$$\begin{array}{l} C_{2}PY_{i}\;=\;\left(MVC_{\left(i-1\right)}13-23\sim MVC_{\left(i\right)}1-12\right)+\left(RED_{\left(i-1\right)}13-23\sim RED_{\left(i\right)}1-12\right)\\ +\left(NIR_{\left(i-1\right)}13-23\sim NIR_{\left(i\right)}1-12\right)+SP_{i}\left[SOS_{i},EOS_{i},\cdots ,LSI_{i},SSI_{i}\right] \end{array}$$

For each C_i_, a random forest (RF) classification was carried out, based on a classifier trained and verified in the reference season (2004/2005). We demonstrated the need of climatic stratification and stratified this national time-series data set according to Zimbabwe's agro-ecological zones.

### Agro-ecological stratification

**Fig 6:**
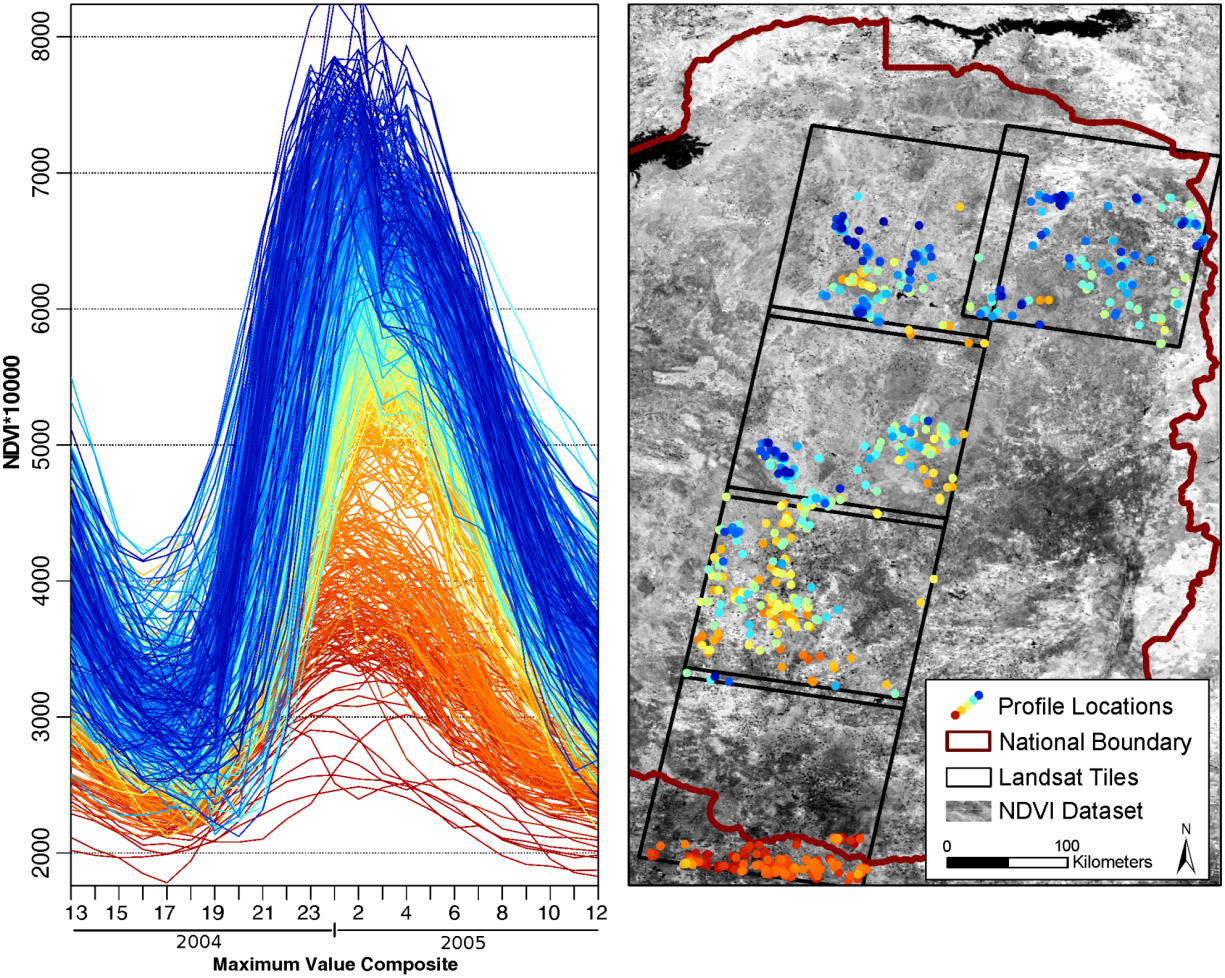
Temporal profiles and spatial location of 750 pure grassland MODIS pixels for phenological season 2004/2005. Color codes represent the maximum value of the NDVI profile. Maximum values follow a South-North increment, related to agro-ecological conditions.

Regional climatic conditions and resulting heterogeneous phenological characteristics form a major limitation for the creation of universal large-area LULC datasets. As discussed above, one restriction of global and continental classifications is the class-definition over different agro-ecological regions which differ in temperature, rainfall, soil type and altitude. Adapted from landscape ecology, where complexity is reduced by breaking areas into patches of similarity [60,61], climatic stratification in other studies has shown to be able to improve multi-temporal classification results [62]. To proof the necessity of a agro-ecological stratification, we constructed 750 endmembers per LULC class following a North-South gradient through Zimbabwe. The endmember construction is described in section 5.3. It became apparent, that similar classes have different seasonal NDVI profiles in different parts of the country, hence preliminary countrywide classifications did not deliver satisfactory results. Fig 6 exemplifies 750 profiles of pure 'grassland' pixels and relates them by color to the North-South gradient. As a consequence of this heterogeneity, we stratified the datasets according to Zimbabwe's agro-ecological zones, restricted the time-series analysis to region AER II and therefore used the endmembers created from Landsat tile 170/72 only. Fig 3 shows the Landsat tiles used for the initial creation of all endmembers for different agro-ecological zones and highlights the tile which was used as input for the final classifications restricted to AER II.

### Endmember construction

To build a training data set of endmembers, a comprehensive classification of Landsat data for season 2004/2005 was carried out. Endmembers are defined as pure MODIS pixels in the training seasons which show a distinctive seasonal curve, representing one LULC class ('rainfed agriculture', 'irrigated agriculture', 'soil', 'grassland', 'woodland'). From the national data set, five Landsat Scenes (170/72-170/75, 169/72; compare Fig 3) were chosen due to their low cloud cover and North-South extent, representing five of six agro-ecological zones across the country. These five scenes were individually processed. To further improve the input dataset for classification, endmembers were stratified according to agro-ecological conditions, as Fig 6 exemplifies its necessity, and their curve separability was improved. Finally, only endmembers of one scene (170/72) were used since we proved that a climatic stratification was necessary and limited the classification to AER II. For this endmember set, an additional LULC class ('sparse rainfed') was introduced after the temporal stratification.

#### Upscaling of Landsat classification

**Fig 7:**
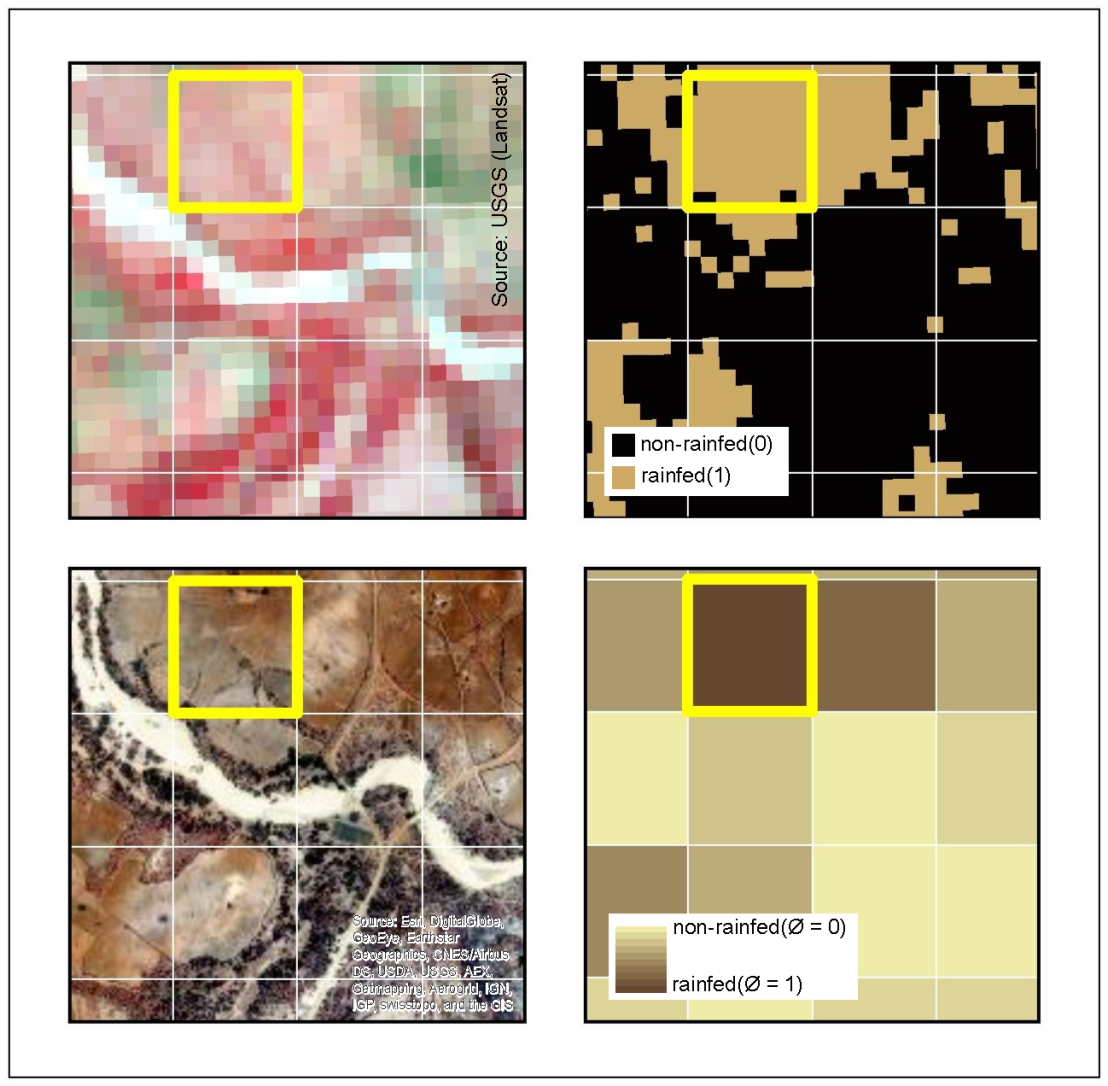
Spatial visualization of endmember construction. Comparison of a pure 'rainfed' endmember (yellow) in Landsat false color image (a), binary classification result for rainfed/other (b), ESRI-basemap (c) and purity grid on MODIS resolution (d).

Without historical on-site knowledge, LULC classes were assigned by interpretation of remote sensing data and the external SADC LULC classification data set. Criteria for training areas were: 1) visual differentiation by shape, greenness and location possible using Landsat imagery, 2) training area within the same class in LULC data set of SADC, 3) shape and location identified in recent high resolution imagery (Bing Maps, Google Earth). If all criteria were met, the feature was considered as a “training area” for the SVM classification. SVM has shown to be able to deliver high accuracies in LULC classifications based on Landsat data and can be used with small training datasets [63]. For the five classified Landsat scenes, overall accuracies ranged between 75% and 85% with high class separability. From these LULC classifications, binary images were produced, which represented one respective class versus all other classes. The inaccuracies of classification were a result of the low separability of grassland, shrubland, and rainfed agriculture which was to be addressed by the multi-temporal analysis. Furthermore, scattered single pixels of different classes occurred throughout the overall solid classifications. To account for the inseparability of classes and scattered pixels, we chose a sampling design which produced pure MODIS pixels with highest accuracy as endmembers. The binary images were resampled to a grid with MODIS resolution of 250 by 250 meters, leading to a purity index for each MODIS pixel. From the polygons with highest purity values, 150 pixels per class were selected for each Landsat tile after they could be manually verified again with the three criteria used for the creation of training data. Fig 7 contrasts the different datasets involved in the selection of pure MODIS training pixels (example visualized in yellow) as endmembers for time-series based classifications.

The upper left part shows an RGB composite of NIR, RED and GREEN bands, where rainfed agriculture is identifiable by its light red color; the MODIS resolution (250x250m) is overlayed as a white grid in all subsets. The upper right detail depicts the same area as the reclassified SVM result. The lower left part shows an ESRI-basemap layer to allow the comparison with data of higher resolution. Finally, the MODIS purity grid is visualized in the lower right part of the figure. To account for difficulties of variable waterbodies, 150 clean pixels were selected manually from an unsupervised classification of NDVI (2004/2005).

#### Improvement of class separability

**Fig 8:**
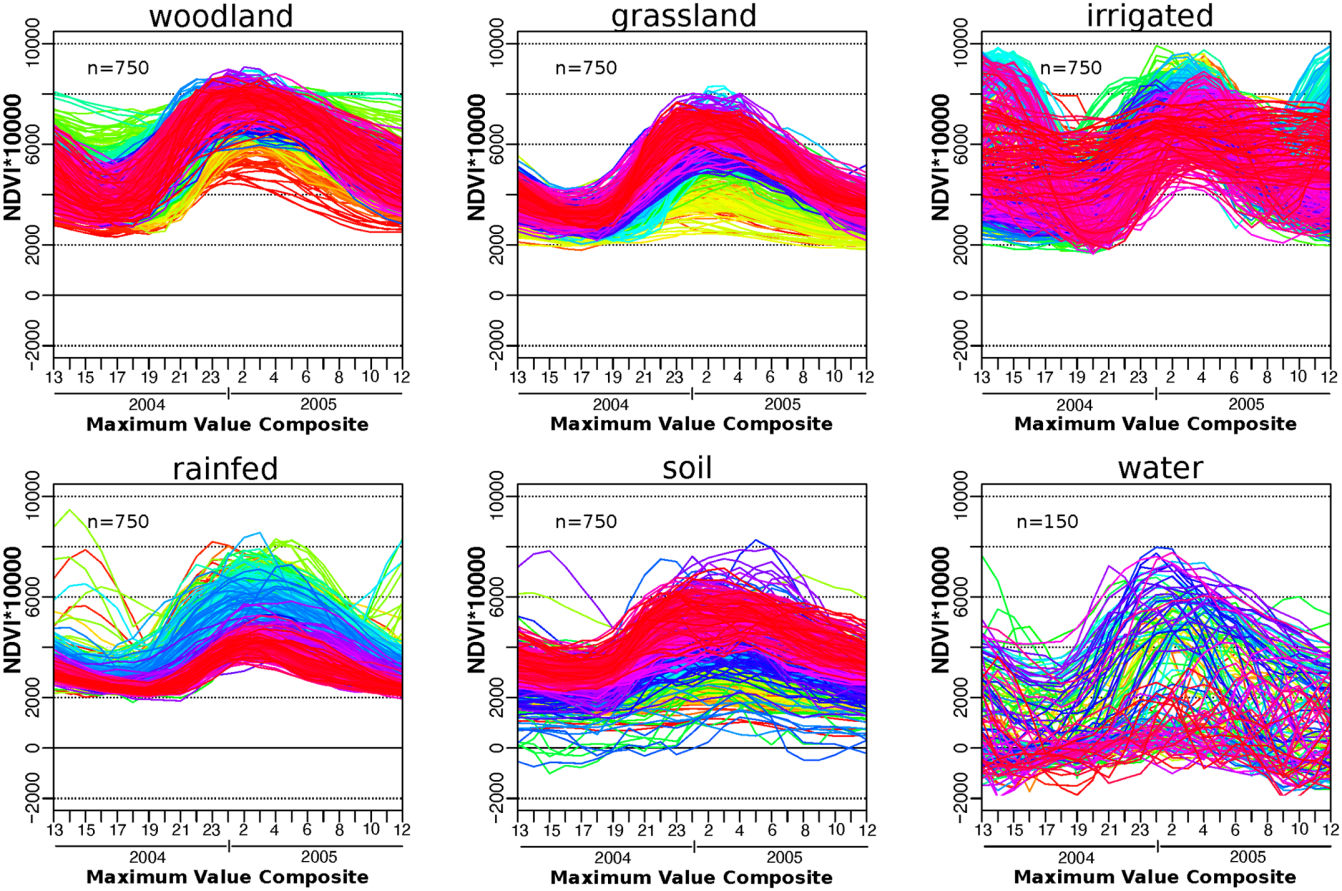
Visualization of heterogeneity of land use classes. Profiles are colored according to longitude. For the selection and verification process of endmember pixels, we made use of two external datasets.

After the selection and verification of 150 pure pixels per class for each of the five Landsat tiles, all 750 temporal NDVI profiles of every LULC were plotted. Fig 8 visualizes NDVI profiles for 750 pixels of each LULC class (water 150) which have been defined as pure pixels according to the endmember construction elaborated previously. From these plots, it becomes evident that the MODIS dataset shows huge variability of temporal NDVI profiles within LULC classes considered as homogeneous according to different datasets which were used to create pure pixels.

**Fig 9:**
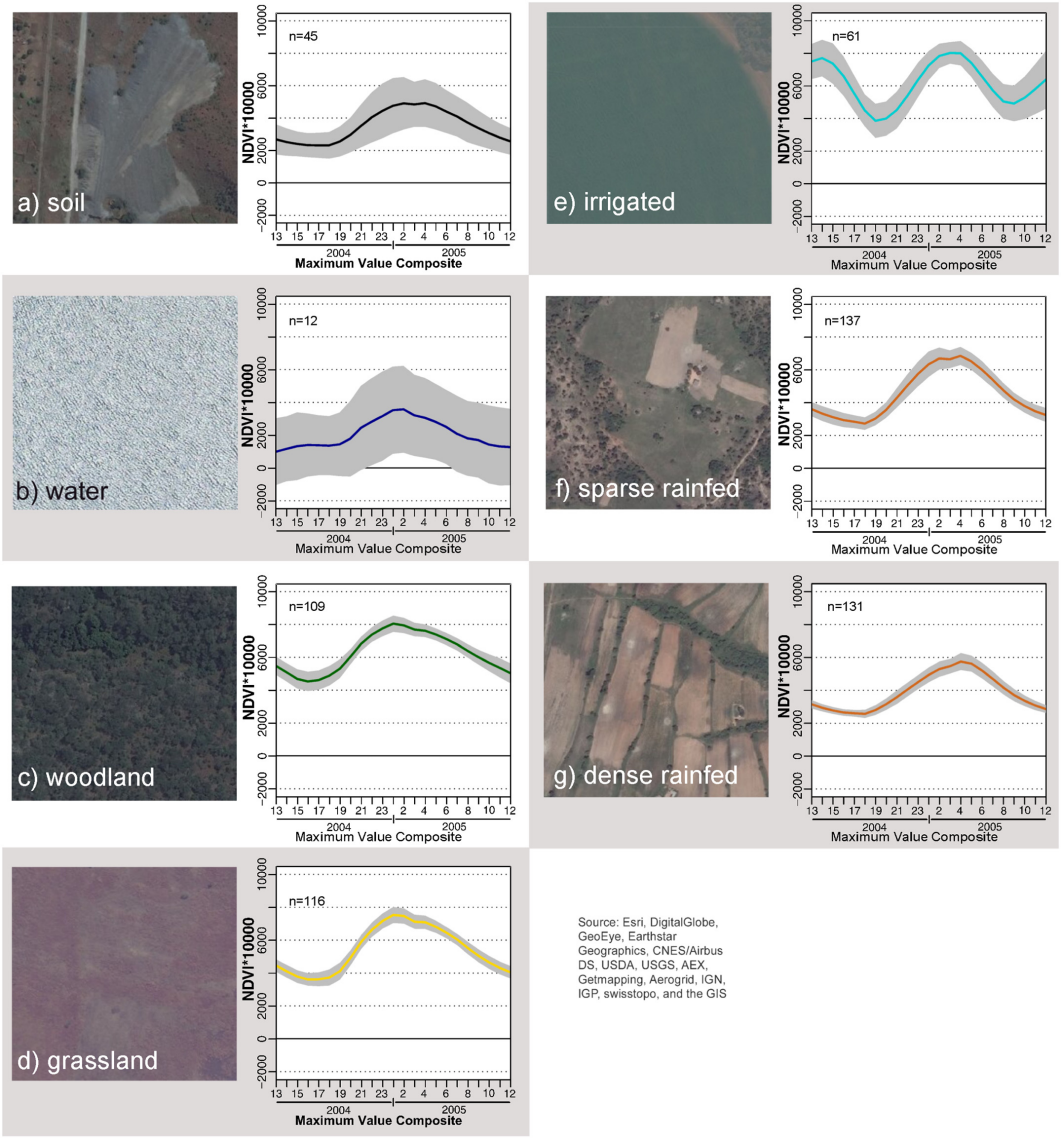
Examples of pure pixels (250x250m) and mean NDVI profiles (2004/2005) including standard deviations for each LULC class.

To create distinct LULC endmembers, temporal profiles were cleaned after the agro-ecological stratification, to generate unique classes with improved separability (Fig 9). To account for differences in the class 'rainfed agriculture', the class was stratified again in 'dense rainfed' and 'sparse rainfed' agriculture. This was done with references from high resolution imagery and unsupervised classification which helped to differentiate the two density types of rainfed agriculture.

### Supervised Random Forest classifications of time-series-data

A classfile was created from endmembers of the seven classes which were created based on Landsat tile 170/72, and formed the basis for subsequent supervised classifications. We made use of imageRF, the random forest (RF) classification implemented in the EnMap-Box. The number of trees was limited to 100, reported as sufficient in other studies and tested in exploratory classifications [64]. The IDL based EnMap-Box provides a convenient environment for supervised classifications such as RF or SVM and was successfully applied in different contexts with similar research designs [65]. Fig 9 correlates mean NDVI as well as standard deviation and a visual pixel example from ESRI-basemap data for each LULC class.

The classes soil (Fig 9a) and water bodies (Fig 9b) show a high variability of NDVI functions although training points were reduced to the most significant. We assume that sparse vegetation for soil and aquatic plants, and siltation for water bodies contribute to the seasonal profile, together with an overall predominant seasonal signal, a side effect of preprocessing in advance of the MOD13Q1 creation. Although this exemplifies difficulties of indifferent reflectance values, it is considered as negligible because differences to the agricultural classes of interest are significant. Studies have proven that RF classification is a suitable method for LULC classification and are capable to separate indifferent classes [66,67].

### Extraction to freehold tenure

The results of the RF classification of climatically stratified time-series were reclassified to binary datasets for each agricultural season to produce datasets of rainfed/non-rainfed area. Because the aim of the research was to test whether MODIS time-series data was able to depict changes in an area of former freehold tenure, we clipped the yearly binary rainfed/non-rainfed datasets to former commercial farms where information on current land tenure was available through the FAS tenure dataset. For this climatically, temporally, and tenure-based stratified classification result, we conducted an accuracy assessment and were therefore able to put focus explicitly on the research question.

## Results and Discussion

The goal of this study was to accurately map the annual extent of rainfed agricultural area between 2001 and 2013 in former commercial farmlands in one specific agro-ecological region in Zimbabwe using MODIS time-series. Our results show spatio-temporal improvements compared to existing regional LULC datasets which, in most cases, are out of date. The additional spectral information (NIR, RED) did not improve RF classification results, as reported by other authors [68]. In order to evaluate the suitability of RF classifications to map rainfed agriculture, reclassified results (rainfed/non-rainfed) were assessed in an error matrix (Tables 2 and 3).

**Table 2:** Accuracy report for NDVI+SP, 2004/2005.

	**rainfed**	**other**	**#pixels**	**UA**
**rainfed**	**95**	55	150	**63,33%**
**other**	3	**147**	150	**98,00%**
**#pixels**	98	202	300	
**PA**	**96,94%**	**72,77%**		**OA = 80,67%**

PA = Producer Accuracy, UA = User Accuracy, OA = Overall Accuracy

**Table 3:** Accuracy report for NDVI+SP,+NIR+RED, 2004/2005.

	**rainfed**	**other**	**#pixels**	**UA**
**rainfed**	**92**	58	150	**61,33%**
**other**	14	**136**	150	**90,67%**
**#pixels**	106	194	300	
**PA**	**86,79%**	**70,10%**		**OA = 76,00%**

PA = Producer Accuracy, UA = User Accuracy, OA = Overall Accuracy

**Fig 10:**
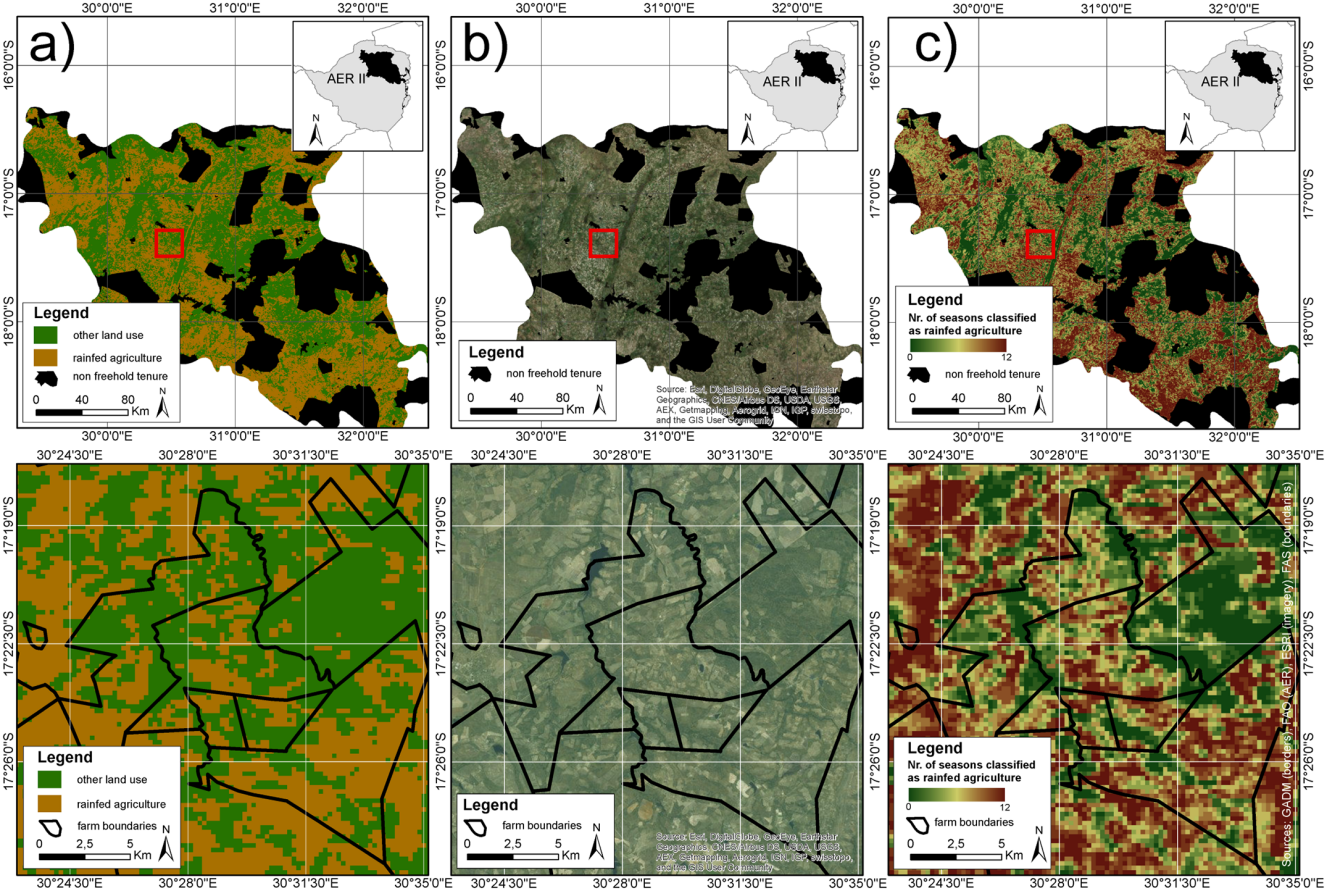
Spatial results of Random Forest classification. (a) classification result (2004/2005) for former freehold tenure area in AER II, (b) recent ESRI-basemap data for comparison, (c) numbers of seasons classified as rainfed or other land use. Details are overlayed with farm boundaries.

Through stratified sampling, 150 pixels for each of the two classes (rainfed/non-rainfed) were selected and compared to historical Google Earth data for the seasons (2004/2005) and (2007/2008) respectively. If more than approximately 75% of the pixel area correlated to the classification result, the pixel was considered as accurate. Google Earth has been used as a verification in other accuracy assessments and provides a convenient method to overcome data unavailability, especially for remote areas on the African continent [69]. Furthermore, it serves as the crucial information independent to the training data. García-Mora et al. emphasize this necessity together with the importance of systematic accuracy assessment with clear protocol. In their review of MODIS based classifications, they conclude that this is often not carried out properly or optimistically biased [70]. Through the selection of the verification dataset, the conservative pixel value of 75% and the grouped report for two classes, we avoid such an optimistic bias. Overall accuracies (OA) for the mapping of rainfed agriculture based on MODIS time-series in the season 2004/2005 ranged from 76% to 80%. Producer accuracies (PA) were high but user accuracies (UA) for 'rainfed' were limited to below 65%. Accuracies for other seasons were within this range. Fig 10a) and 10c) contrast the classification result based on NDVI data for season 2004/2005 with recent ESRI-basemap imagery (Fig 10b). The upper row of maps show AER II clipped to former freehold tenure, the lower row of maps depicts a detail overlayed with commercial farm boundaries. Here, we observe the underestimation of agricultural fields which links to the low user accuracies of the class rainfed agriculture. This is a critical error because it limits the spatially-explicit assessment of land reform effects. Changes in cropping patterns are likely to not be recognized by the classification approach, especially because they appear at the edges of LULC patches. This is demonstrated in Fig 10c) which summarizes classification results of all twelve consecutive seasons from 2001-2013 and therefore identifies areas which are continuously classified as 'rainfed agriculture' and areas which are considered as 'other' throughout. In-between these stable land use patches, areas were not continuously classified as one land use type. This might either be related to a sensitive classifier, or a systematic error resulting from low separability of sparse rainfed agriculture. But with class accuracies below 65%, the volatility cannot be considered to be a consequence of changes in land ownership in former freehold tenure area. Inconsistent land use classification occurs only at the edges of stable areas and does not depict agricultural patches. This temporal stack might be used to improve general land use classifications, but it does not allow a linkage of cropping pattern and land tenure.

Although time-series as a method has proven to be able to depict general LULC patterns over time, and change models as well as trajectories have been applied successfully in the region [71], our evaluation demonstrates the limitations of the method. The difficulties of correctly mapping rainfed area, are related to shortcomings which have been formulated by other authors [35,71–73]. Spatially-accurate crop mapping remains a bottleneck in multi-temporal analysis, although we have produced higher accuracies than studies in different contexts [62].

We identified two major limitations of multi-temporal analysis of MODIS NDVI data to map commercial farmland structures in Zimbabwe: *1) Spatial resolution* of imagery and *2) heterogeneity* of spatiotemporal profiles. Alongside successful studies, which also have characterized Zimbabwean smallholder farming structures of sizes below MODIS resolution [16], authors report high inaccuracies for detection of smaller LULC patches such as heterogeneous, sparse cropland in other study areas [62,73]. Low user accuracies, combined with high producer accuracies such as in this analysis, can be sufficient for general LULC classifications with several classes. For the spatial explicit mapping of one specific land-use class and its spatial correlation with land tenure, they are not adequate. Our accuracy assessment was designed to test the ability of regional time-series analysis to map crop area in former freehold tenure, because its sparse and heterogeneous patterns differ from rainfed agriculture in communal areas. The communal form of land use is characterized by dense fields with distinct reflectance values and is therefore clearly recognized in classifications and has received attention in other studies [74,75]. With the difficulties to map sparse commercial farm structures, we conclude that coarse-scale spatio-temporal analysis is not suitable to generate well-founded knowledge of change patterns for cultivated areas as a consequence of tenure change. Also regional assessments of spatio-temporal land reform effects should therefore be carried out on smaller scale with high resolution imagery, which was applied somewhat successfully [76,77]. Current approaches in Zimbabwe which aim to map the abandonment of dams as part of an intact irrigation system are another possible solution.

Overall, we experienced shortcomings of regional assessment based MODIS data which we identified for global land-cover mapping earlier. Our plots of NDVI functions of training pixels, on a country-wide basis (Fig 8), as well as the stratified sample within the AER II (Fig 9), demonstrate the difficulties to assign classes with distinct and homogeneous temporal profiles. Even after agro-ecological and temporal stratification, which limited training pixels to a small and very distinct number, the plotted standard deviations demonstrate that unique classes cannot be defined based on temporal profiles. This has also been formulated for large scale, global and continental classifications [72,78,79].

Because MODIS multi-temporal data is not able to accurately classify sparse former commercial farmland on a spatial resolution which allows a correlation with land tenure data, we propose a different approach: Vegetation productivity and trend analysis. Both can be determined based on NDVI time-series and are methods which determine relative parameters instead of a hard classification into different LULC classes. They therefore avoid inseparability of classes and sub-pixel heterogeneity which we identified as the major limitations of medium-resolution, high temporal imagery. Productivity and trend analysis have been used extensively in the context of agricultural mapping, also in Zimbabwe [48,75,80]. In South Africa, these methods have also been applied to assess the effect of unequal land tenure and population density [74]. A synergistic research design of a productivity trend analysis and our agro-ecological and tenure-based stratification would allow to assess the condition of redistributed commercial farmland in Zimbabwe.

## Conclusion

Spatial datasets on agricultural land are crucial in order to understand the intensively debated process of the FTLRP of Zimbabwe as well as its consequences. The overall research question of this methodological assessment was whether multi-temporal LULC classification is able to produce spatially accurate maps of rainfed cropping area to determine changes in agricultural production as a consequence of redistribution of farmland in Zimbabwe. To answer this question, MODIS NDVI and spectral data was smoothed with a Savitzky-Golay filtering approach, temporally and climatically stratified and limited to former freehold tenure. Endmembers were created by an upscaling approach of Landsat classification. Random forest classifications of time-series based on the stratified endmembers showed higher accuracies than existing global and regional LULC products and similar studies. General distribution of LULC classes were depicted using the RF classifier. However, the classification results cannot be used in the context of an assessment of land reform in Zimbabwe. Because commercial farms form fragmented agricultural landscapes, they cannot be accurately depicted by MODIS time-series and are often underestimated and confused with grassland.

Our research with a focus on coupling of land tenure and land use has revealed major shortcomings of regional classification approaches and MODIS MOD13Q1 NDVI data. 1) regional classifications stretch over climatic gradients which leads to large differences in the phenological cycle of one land-cover class making the creation of homogeneous classes impossible. Even within similar agro-ecological conditions, a high variability of temporal NDVI profiles persists within classes. 2) spatial heterogeneity, combined with the resolution of MODIS and class inseparability, leads to inter-annual inaccuracies of time-series based land-cover classification decisions. Classification results aggregated over several seasons show a high variability at land-use patches highlighting the likeliness of wrong allocation. 3) These shortcomings weaken mapping of land-cover change based on multi-temporal classifications, and highlight the necessity of detailed information on the ground. Given the fact that land-use, especially changes of cropping patterns, are frequent products of immediate socio-economic decisions, the volatile multitemporal products do not allow to draw conclusions whether and when a change of land use has occurred over the course of several years. As a consequence, the information of moderate-resolution is not able to be linked to possible socio-economic drivers of land-cover change such as ownership or management change due to a land reform program. However, long sequences of several continuous classifications could provide useful input to land use classification by providing information of probability and confidence.

As time-series analysis as a valuable and approved method has difficulties to detect LULC types and change in an area of interest for a FTLRP assessment, we propose to use this methodology for a vegetation trend and productivity analysis among different land tenure types. It should be tested whether MODIS data is capable to differentiate productivity trends in redistributed farmland and therefore be able to allow a comparative assessment of the impact of farmland redistribution and the role of land tenure.
